# Intrinsic Fluctuations in Transpiration Induce Photorespiration to Oxidize P700 in Photosystem I

**DOI:** 10.3390/plants9121761

**Published:** 2020-12-12

**Authors:** Riu Furutani, Amane Makino, Yuij Suzuki, Shinya Wada, Ginga Shimakawa, Chikahiro Miyake

**Affiliations:** 1 Graduate School for Agricultural Sciences, Kobe University, 1-1 Rokkodai, Nada, Kobe 657-8501, Japan; 201a521a@stu.kobe-u.ac.jp (R.F.); swada@penguin.kobe-u.ac.jp (S.W.); 2 Core Research for Environmental Science and Technology, Japan Science and Technology Agency, 7 Gobancho, Tokyo 102-0076, Japan; amanemakino@tohoku.ac.jp (A.M.); ysuzuki@iwate-u.ac.jp (Y.S.); gshimakawa@rcsec.chem.es.osaka-u.ac.jp (G.S.); 3 Graduate School of Agriculture Science, Tohoku University, 468-1 Aramaki Aza Aoba, Aoba, Sendai 980-8572, Japan; 4 Faculty of Agriculture, Iwate University, 3-18-8 Ueda, Morioka, Iwate 020-8550, Japan; 5 Research Center for Solar Energy Chemistry, Osaka University, 1-3 Machikaneyama, Toyonaka, Osaka 560-8631, Japan

**Keywords:** P700, photorespiration, photosystem I, ΔpH, RISE

## Abstract

Upon exposure to environmental stress, the primary electron donor in photosystem I (PSI), P700, is oxidized to suppress the production of reactive oxygen species that could oxidatively inactivate the function of PSI. The illumination of rice leaves with actinic light induces intrinsic fluctuations in the opening and closing of stomata, causing the net CO_2_ assimilation rate to fluctuate. We examined the effects of these intrinsic fluctuations on electron transport reactions. Under atmospheric O_2_ conditions (21 kPa), the effective quantum yield of photosystem II (PSII) (Y(II)) remained relatively high while the net CO_2_ assimilation rate fluctuated, which indicates the function of alternative electron flow. By contrast, under low O_2_ conditions (2 kPa), Y(II) fluctuated. These results suggest that photorespiration primarily drove the alternative electron flow. Photorespiration maintained the oxidation level of ferredoxin (Fd) throughout the fluctuation of the net CO_2_ assimilation rate. Moreover, the relative activity of photorespiration was correlated with both the oxidation level of P700 and the magnitude of the proton gradient across the thylakoid membrane in 21 kPa O_2_ conditions. These results show that photorespiration oxidized P700 by stimulating the proton gradient formation when CO_2_ assimilation was suppressed by stomatal closure.

## 1. Introduction

As photosynthetic organisms have evolved, the close coupling between the light reaction and the Calvin–Benson–Bassham (CBB) cycle has strengthened [1]. In the light reaction of C3 plants, both photosystem I (PSI) and photosystem II (PSII) absorb light energy from the sun and drive the photosynthetic linear electron flow from H_2_O to NADPH. PSII oxidizes 2H_2_O to O_2_ and 4H^+^ to extract electrons and protons. The electrons flow to PSI through plastoquinone (PQ), the cytochrome (Cyt) *b*_6_*f* complex, and plastocyanin (PC). PSI catalyzes the electrons transferred from the reduced PC to ferredoxin (Fd). In this process, electrons flow from the lumen to the stroma across the thylakoid membranes. Finally, the reduced Fd donates electrons to NADP^+^, producing NADPH. With this linear electron flow, H^+^ accumulates in the thylakoid lumen as a result of both H_2_O oxidation by PSII and the movement of protons from the stroma to the lumen during the oxidation of PQ by the Cyt *b*_6_*f* complex. The accumulated H^+^ drives the production of ATP by ATP synthase, which is embedded into the thylakoid membrane. Therefore, the light reaction produces two chemical energy compounds, NADPH and ATP. The CBB cycle of C3 plants is driven by both CO_2_ assimilation and photorespiration simultaneously, using both the NADPH and the ATP produced in the light reaction. Both compounds are used for the regeneration of ribulose 1,5-bisphophate (RuBP), which is one of the substrates of RuBP carboxylase/oxygenase (Rubisco). The electron flux in the linear electron flow of the light reaction is almost equal to the flux of electrons consumed in the CBB cycle [2,3,4].

Photorespiration, which drives the CBB cycle with CO_2_ assimilation, starts with the oxygenation of RuBP to 3-phosphoglycerate (PGA) and 2-phosphoglycolate (PG) catalyzed by Rubisco. To regenerate PGA from PG, a series of reactions is carried out via the peroxisomes and mitochondria, releasing CO_2_ and NH_3_. During this process, additional ATP and reduced products from the light reaction (Fd) are used for the regeneration of PGA and the re-assimilation of NH_3_ [5]. These reactions seem to be wasteful and the physiological role of photorespiration is still under debate. However, it has been suggested that photorespiration acts as a strong electron sink and plays an important role in the dissipation of excess light energy in the photosynthetic electron transport system [1,6,7,8,9,10,11].

The coupling between the light reaction and the CBB cycle requires an interaction between their systems to suppress the production of reactive oxygen species (ROS) in PSI [2]. For example, saturated pulse illumination (5,000–20,000 µmol photons m^−2^ s^−1^ of light intensity, 300–800 ms duration) in the dark causes the rapid accumulation of electrons at the acceptor side of PSI [12,13]. Unless these electrons are consumed in the CBB cycle, they will accumulate in the photosynthesis electron transport system and flow to O_2_ via the univalent reduction of O_2_ to the superoxide anion radical (O_2_^−^). The O_2_^−^ then spontaneously and rapidly disproportionates to hydrogen peroxide (H_2_O_2_) and O_2_ [14,15]. These ROS oxidatively inactivate PSI function [12]. Therefore, suppression of the CBB cycle can result in an accumulation of electrons and the production of ROS in the photosynthesis electron transport system.

The selective photoinhibition of PSI caused by ROS has been observed in specific situations [16,17,18], which shows that all oxygenic photosynthetic organisms have a molecular mechanism to suppress the production of ROS in PSI. It is well established that ROS can be produced in PSI [19]. However, it is not yet fully understood how ROS production in PSI is suppressed [20,21,22]. Miyake et al. described a P700 oxidation system to suppress the production of ROS in PSI [12,23]. The photo-oxidation/reduction cycle of P700—the primary electron donor of PSI—drives the electron flow from PC to Fd. Illumination with actinic light excites P700 to P700* (excited P700). The P700* donates an electron to electron carriers localized at the acceptor side of PSI for the reduction of Fd and then NADP^+^. Simultaneously, P700* is oxidized to P700^+^, then the P700^+^ is reduced by the reduced PC to the ground state of P700. Thus, the production of ROS is regulated by the redox state of P700: if P700* oxidation is rate-limited, electrons accumulate at the acceptor-side of PSI and flow toward ROS production. Conversely, if P700^+^ reduction is rate limited, O_2_ reduction is suppressed and electron accumulation at the acceptor-side of PSI is relieved [2,24]. When the efficiency of CO_2_ assimilation is reduced, such as under high light intensity, low CO_2_ concentrations, drought, or extreme temperatures, all oxygenic photosynthetic organisms can oxidize P700 and suppress ROS production [13,25,26,27]. That is, the P700^+^ reduction reaction becomes rate-determining, and the accumulation of P700^+^ suppresses the accumulation of P700*, which decreases the likelihood of ROS production in PSI.

All oxygenic photosynthetic organisms have evolved P700 oxidation systems [2,13]. As mentioned above, the transition of the rate-determining steps in the P700 photo-oxidation/reduction cycle is key to maintaining P700 in the oxidized state. Under environmental conditions that reduce the efficiency of CO_2_ assimilation, the P700^+^ reduction reaction becomes rate-determining to keep P700 oxidized. Electron flux in the photosynthetic electron transport system can also be controlled via the down-regulation of the functions of both PSII and the Cyt *b*_6_*f* complex [2,28]. H_2_O oxidation in PSII is down-regulated by lumen acidification, which also triggers the non-photochemical quenching (NPQ) of chlorophyll (Chl) fluorescence [29]. The oxidation of reduced PQ, which is catalyzed by the Cyt *b*_6_*f* complex, is down-regulated by both lumen acidification [30,31] and the reduction-induced suppression of electron flow [32,33]. Suppression of the CO_2_ assimilation efficiency also induces lumen acidification, increasing both the proton motive force (pmf), which consists of the proton gradients across the thylakoid membranes (ΔpH) and other ion gradients (ΔΨ), and levels of the reduced form of PQ [11,13]. The pmf is determined by the ratio of the H^+^ consumption rate (JgH^+^) to the H^+^ conductance (gH^+^) [34,35]. The reduction/oxidation ratio of PQ is determined by the ratio of electron flux in the photosynthetic linear electron flow (Jf) to the apparent rate constant (kqL) for the oxidation of reduced PQ by the Cyt *b_6_f* complex [2,24]. The gH^+^ reflects the efficiency of both ADP and Pi regeneration and ATP synthase activity; lower photosynthetic activity leads to increased pmf [2,26]. The accumulation of reduced PQ suppresses the Q-cycle activity of the Cyt *b*_6_*f* complex [2,24,32,33]. All of these regulatory mechanisms contribute to keeping P700 in the oxidized form upon exposure to CO_2_ assimilation-limited photosynthesis conditions [2]. 

In the present study, we examined the effects of intrinsic fluctuations in the net CO_2_ assimilation rate on the regulation of P700 oxidation in PSI of rice plants. Fluctuations in the rate of net CO_2_ assimilation occur when the stomata open and close. Rice plants exhibit fluctuations in their transpiration rate with random frequency, which we cannot control. Therefore, we can expect fluctuations in the electron sink activity of CO_2_ assimilation. We analyzed how P700 is oxidized in response to these fluctuations in CO_2_ assimilation efficiency. We found that the intrinsic fluctuations in transpiration caused by the stomata opening and closing suppressed the net CO_2_ assimilation rate when the stomata were closed, and induced photorespiration. This led to oxidization of P700 by the formation of a proton gradient across the thylakoid membranes and suppression of ROS production in PSI. We discuss the molecular mechanism of P700 oxidation during the fluctuations in the net CO_2_ assimilation rate. 

## 2. Results

### 2.1. Effects of Fluctuations in the Net CO_2_ Assimilation Rate on the Effective Quantum Yield of PSII (Y(II)) and Photorespiration Rate

**Figure 1:**
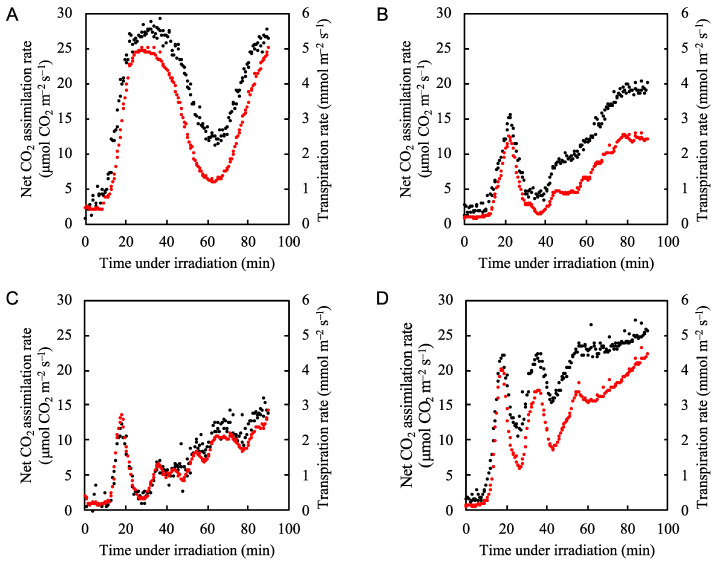
Fluctuation patterns of both the net CO_2_ assimilation rate and transpiration rate under the steady-state actinic light illumination (700 µmol photons m^–2^ s^–1^) in rice leaves. Four examples are shown (**A–D**). The net CO_2_ assimilation rate (black circles) and transpiration rate (red circles) were measured under 21 kPa O_2_/40 Pa CO_2_ conditions. Actinic light was turned on at 0 min.

We first measured the net CO_2_ assimilation and transpiration rates over time after application of actinic light to rice leaves under normal atmospheric conditions. In Figure 1, we show the four examples of the net CO_2_ assimilation rate and transpiration rate under the steady light conditions (Figure 1A–D). Upon illumination with actinic light (700 µmol photons m^−2^ s^−1^), the net CO_2_ assimilation rate gradually increased, with several minutes of lag time (Figure 1A–D). This was due to the stomata opening, as indicated by the increase in transpiration rate (Figure 1A–D). The net CO_2_ assimilation rate fluctuated in a somewhat random manner, as indicated by the various patterns shown in Figure 1. The fluctuations in the net CO_2_ assimilation rate were harmonized with those of the transpiration rate (Supplementary Figure S1). We could not regulate the fluctuation frequency of the net CO_2_ assimilation rate. Therefore, in the following experiments all analyses were performed under conditions where the net CO_2_ assimilation rate fluctuated along with the transpiration rate. In the following sections, we show representative data for one individual plant and data from all parameters for another two individuals are shown in the Supplementary Materials. We mainly discuss results according to the representative data, confirming that the same tendencies described here were observed in each measurement.

**Figure 2:**
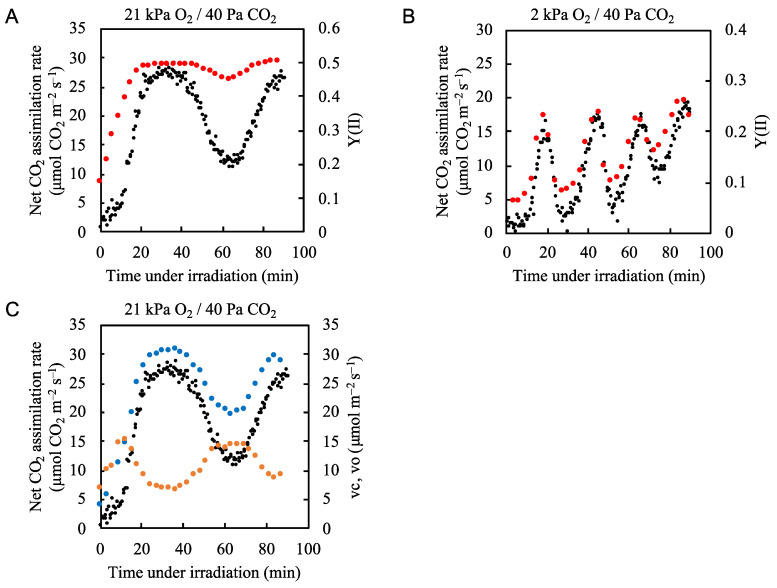
Effects of partial pressure of O_2_ on both the net CO_2_ assimilation rate and quantum yield of PSII (Y(II)) in rice leaves. The net CO_2_ assimilation rate (black circles) and Y(II) (red circles) were simultaneously measured under (**A**) 21 kPa O_2_/40 Pa CO_2_ conditions and (**B**) 2 kPa O_2_/40 Pa CO_2_ conditions. Actinic light (700 µmol photons m^–2^ s^–1^) was turned on at 0 min. The data for the net CO_2_ assimilation rate at 21 kPa O_2_/40 Pa CO_2_ conditions are the same as those used for Figure 1A. (**C**) Both ribulose 1,5-bisphophate (RuBP) carboxylation rate (vc) (blue circles) and RuBP oxygenation rate (vo) (orange circles) were estimated by the method, described in Materials and Methods. Both vc and vo as well as the net CO_2_ assimilation rate (black circles) are plotted against the time after illumination of actinic light.

Upon illumination with actinic light under conditions of 21 kPa O_2_/40 Pa CO_2_, which closely resemble normal atmospheric conditions at sea level, the net CO_2_ assimilation rate gradually increased, while the effective quantum yield of PSII (Y(II)) rapidly increased and reached a peak value before the net CO_2_ assimilation rate peaked (Figure 2A, Supplementary Figure S4). This indicates that we observed an increased electron flux in PSII, which was not driven by the CO_2_ assimilation; thus, an alternative electron flow was functioning. Sejima et al. and Hanawa et al. [1,10] showed that photorespiration begins as soon as actinic light is applied to the leaves of C3 plants. Therefore, the extra electron flux in PSII, which did not follow the increase in the net CO_2_ assimilation rate, was most likely driven by photorespiration. Furthermore, while the net CO_2_ assimilation rate fluctuated, the extra electron flux in PSII remained near its maximum (Figure 2A). This suggests that photorespiration maintained the electron flux in PSII despite the changes in net CO_2_ assimilation rate.

We next tested the hypothesis that photorespiration functions as the alternative electron flow mechanism when CO_2_ assimilation does not occupy the main electron flux in PSII. We decreased the partial pressure of O_2_ from 21 to 2 kPa to suppress the oxygenase activity of RuBP carboxylase/oxygenase [11,36]. The electron flux in PSII, as shown by Y(II), followed the fluctuation in the net CO_2_ assimilation rate (Figure 2B, Supplementary Figure S4). This indicates that under atmospheric conditions (21 kPa O_2_/40 Pa CO_2_) photorespiration was the main alternative electron flow. 

We estimated both the RuBP carboxylation rate (vc) and the RuBP oxygenation rate (vo) based on the net CO_2_ assimilation rate and the effective quantum yield of PSII [1,10,11,37,38] (Figure 2C). Upon illumination with actinic light, the vo rapidly increased to approximately 15 µmol O_2_ m^−2^ s^−1^, then decreased to about 7 µmol O_2_ m^−2^ s^−1^ about 35 min after the start of illumination. At the same time, the vc increased and reached a maximal value of approximately 30 µmol CO_2_ m^−2^ s^−1^ at 35 min. After 40 min of illumination, both the net CO_2_ assimilation rate and the vc began to decrease, reaching minimal values after 60 min. The pattern of change in vo contrasted with the vc and the photosynthetic rate, reaching its minimal value at 35 min and its maximal value at 60 min. These results suggest that photorespiration compensated for the decrease in the electron sink when CO_2_ assimilation was suppressed.

As shown in Figure 2B, under 2 kPa O_2_ conditions the fluctuations in Y(II) closely resembled the fluctuations in net CO_2_ assimilation rate. However, at each minimum value, the Y(II) values were from 0.02 to 0.05 higher than would be expected if the Y(II) followed the photosynthetic rate more closely (Figure 2B). We plotted the electron transport rate (ETR) at PSII against four-times the net CO_2_ assimilation rate under 2 kPa O_2_ conditions because 4 mol electrons are needed to assimilate 1 mol CO_2_, and we found an excessive electron flux of about 10 µmol e^–^ m^−2^ s^−1^ (Supplementary Figure S2). These higher values most likely reflect the electron fluxes of N, S-assimilation, mitochondrial respiration [39,40], and/or the water-to-water cycle [41].

### 2.2. Effects of Fluctuations in Net CO_2_ Assimilation Rate on the Redox States of PC, P700, Fd, and the PQ Pool 

**Figure 3:**
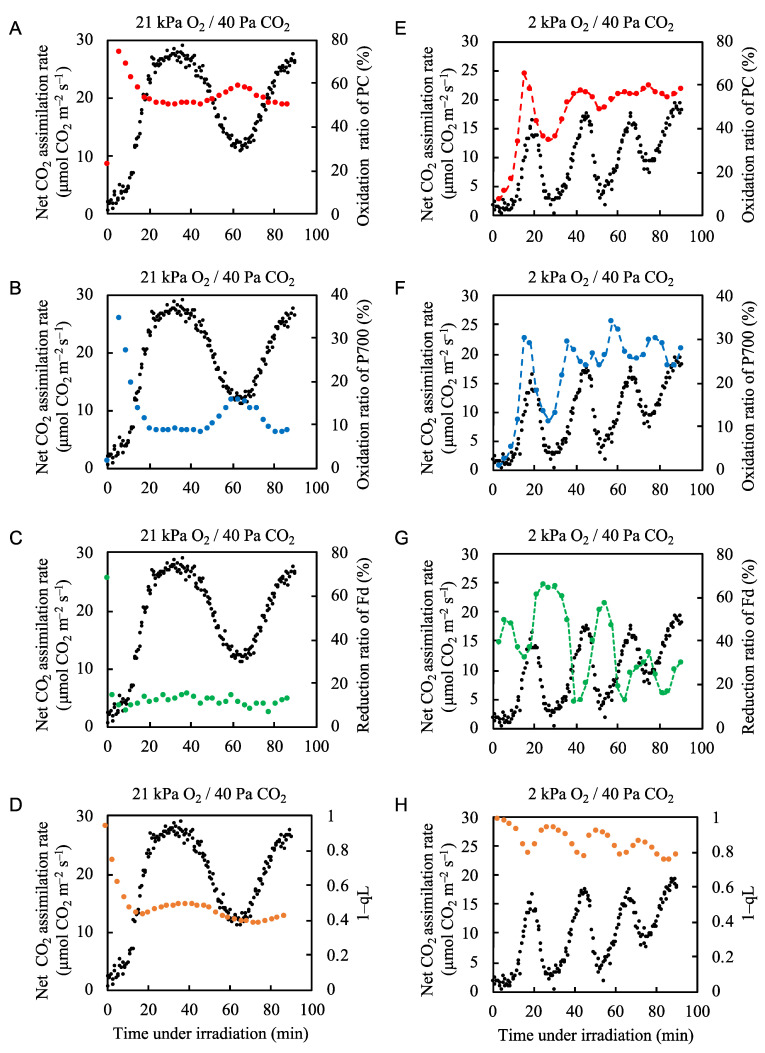
Effects of the partial pressure of O_2_ on the net CO_2_ assimilation rate and the redox states of plastocyanin (PC), P700, ferredoxin (Fd), and plastoquinone in rice leaves. The oxidation ratios of PC (red circles) and P700 (blue circles), and the reduction ratios of Fd (green circles) and plastoquinone (1-qL) (orange circles) were measured simultaneously with the net CO_2_ assimilation rate (black circles) under 21 kPa O_2_/40 Pa CO_2_ conditions (**A–D**) and 2 kPa O_2_/40 Pa CO_2_ conditions (E-H). Actinic light (700 µmol photons m^–2^ s^–1^) was turned on at 0 min. The data for the net CO_2_ assimilation rate at 21 kPa O_2_ are the same as used for Fig. 1A and the data for the net CO_2_ assimilation rate at 2 kPa O_2_ are the same as those used for Figure 2B. Dashed lines in the graph (**E–G**) was arbitrarily drawn to indicate the trend of the data.

We showed that despite the intrinsic fluctuations in the net CO_2_ assimilation rate, the electron flux in PSII is maintained because photorespiration is activated when CO_2_ assimilation is suppressed (Figure 1 and Figure 2). Generally, decreases in the net CO_2_ assimilation rate induce the oxidation of P700 to suppress the production of ROS in PSI [2,12]. We next examined the effects of fluctuations in the net CO_2_ assimilation rate on the redox states of PC, P700, Fd, and the PQ pool under normal and low O_2_ conditions (Figure 3, Supplementary Figures S5–S8). Under conditions of 21 kPa O_2_/40 Pa CO_2_, illumination with actinic light caused the oxidation ratios of PC and P700 to rapidly increase and then decrease as the net CO_2_ assimilation rate increased (Figure 3A,B). The oxidation ratios of PC and P700 then remained constant while the photosynthetic rate increased, reached its maximal level, and began to decrease, and they showed relatively small peaks of increased oxidation that coincided with the minimal net CO_2_ assimilation rate. Thus, the oxidized states of both PC and P700 showed lower values when the net CO_2_ assimilation rate was high. Conversely, the redox state of Fd did not respond to fluctuations in the net CO_2_ assimilation rate, except for a rapid switch to the reduced state at the onset of illumination, before rapidly returning to a mainly oxidized state (Figure 3C). These results correspond with those of Kadota et al. [42]. The Chl fluorescence parameter 1–qL, which reflects the reduction level of the primary electron acceptor of PSII, Q_A_, also shows the reduction ratio of the PQ pool. Here, we assumed a rapid equivalization between the Q_A_ site in PSII and the PQ-pool [43,44,45]. Illumination with actinic light caused a rapid reduction of the PQ pool, followed by a rapid return to a more oxidized state (Figure 3D). Thereafter, the reduction ratio of the PQ pool showed slight increases and decreases that followed the pattern of the net CO_2_ assimilation rate (Figure 3D). 

Under normal atmospheric conditions (21 kPa O_2_/40 Pa CO_2_), we identified photorespiration as the main alternative electron flow mechanism when the net CO_2_ assimilation rate fluctuated (Figure 2). To elucidate the effects of photorespiration on the redox states of PC, P700, Fd, and the PQ pool, we decreased the partial pressure of O_2_ from 21 to 2 kPa to suppress photorespiration in the rice plants [46].

When photorespiration was suppressed at 2 kPa O_2_, the application of actinic light did not induce the rapid oxidation of PC and P700 (Figure 3E,F). Both PC and P700 became more oxidized with increases in the net CO_2_ assimilation rate and their levels of oxidation followed the fluctuations in the net CO_2_ assimilation rate (Figure 2B). The redox states of Fd and the PQ pool followed the same pattern, with their reduction ratios mirroring the fluctuations in the net CO_2_ assimilation rate (Figure 3G,H). In particular, the reduction state of Fd appeared to be almost exclusively driven by the photosynthetic linear electron flow, as was the electron flux in PSII (Figure 2B). These results correspond to those of Kadota et al. [42].

**Figure 4:**
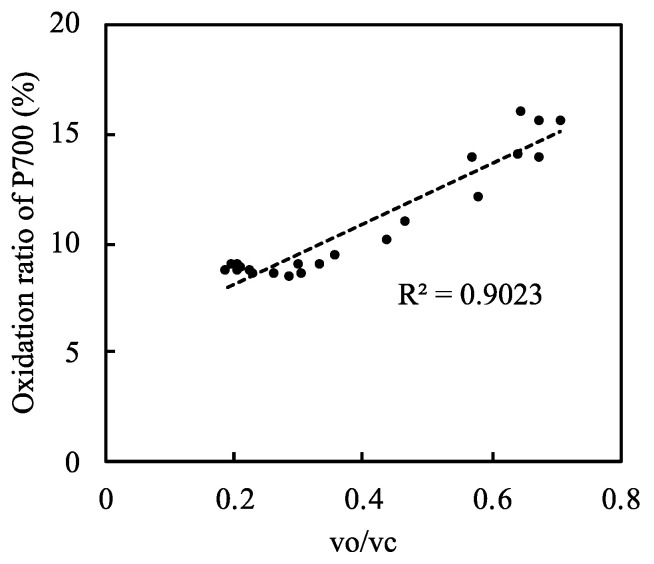
Relationship between the relative photorespiration activity (vo/vc) and the oxidation ratio of P700 in rice leaves. Both ribulose 1,5-bisphophate (RuBP) carboxylase reaction rate (vc) and RuBP oxygenase reaction rate (vo) were estimated by the method described in Materials and Methods. Data from Figure 2C and Figure 3B were used. Dashed line in the graph was the regression line and the coefficient of determination was 0.9023 (*p* < 0.001).

Under normal atmospheric conditions, photorespiration functions as an alternative electron sink and drives the photosynthetic linear electron flow [11]. Therefore, photorespiration contributes to the oxidized states of PC, P700, Fd, and PQ. To further examine the relationship between P700 oxidation and photorespiration, we plotted the oxidation level of P700 against the vo/vc ratio (Figure 4, Supplementary Figure S9). As the vo/vc ratio increased, the oxidation of P700 increased; this occurred when the net CO_2_ assimilation rate was low (Figure 2B). Thus, photorespiration induced the oxidation of P700 by driving the linear electron flow when the CO_2_ assimilation was suppressed, keeping the electron flux nearly constant.

### 2.3. Effects of Fluctuations in the Net CO_2_ Assimilation Rate on the Non-Photochemical Quenching of Chl Fluorescence and the Formation of the Proton Gradient (∆pH) across the Thylakoid Membrane

**Figure 5:**
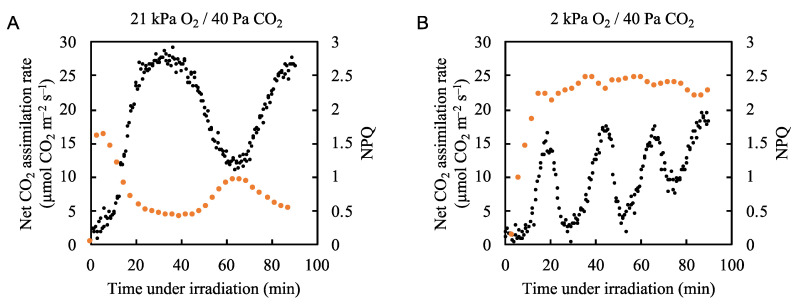
Effects of partial pressure of O_2_ on the relationship between the net CO_2_ assimilation rate and non-photochemical quenching (NPQ) of chlorophyll fluorescence in rice leaves. NPQ of chlorophyll fluorescence (orange circle) was measured simultaneously with the net CO_2_ assimilation rate (black circles) under (**A**) 21 kPa O_2_/40 Pa CO_2_ and (**B**) 2 kPa O_2_/40 Pa CO_2_ conditions. Actinic light (700 µmol photons m^–2^ s^–1^) was turned on at 0 min. The data for the net CO_2_ assimilation rate under 21 kPa O_2_/40 Pa CO_2_ and 2 kPa O_2_/ 40 Pa CO_2_ are the same as those used for Figure 2A and 2B, respectively.

To understand the molecular mechanism by which photorespiration induces P700 oxidation in PSI, we analyzed the effects of fluctuations in the net CO_2_ assimilation rate on the NPQ of both Chl fluorescence and ∆pH (with lumen acidification) across the thylakoid membrane. Under atmospheric conditions, the rate of NPQ followed the same pattern as P700 oxidation (Figure 3B and Figure 5A, Supplementary Figures S7 and S10). Upon illumination with actinic light, NPQ was rapidly induced and started to decrease to a steady state as the net CO_2_ assimilation rate increased. Like the P700 oxidation ratio, the NPQ showed a relatively small peak when the net CO_2_ assimilation rate was at its minimum. Under low oxygen conditions (2 kPa O_2_/40 Pa CO_2_), NPQ increased to a relatively high value of 2.3 upon actinic light illumination and then showed minor fluctuations, with the highest level of quenching occurring when the net CO_2_ assimilation rate was lowest (Figure 5B, Supplementary Figure S10). These results suggest that photorespiration was not responsible for the enhancement of the ΔpH formation to oxidize P700. However, it is difficult to understand what the NPQ value really reflected under 2 kPa O_2_ conditions because Y(II) fluctuated strongly (Figure 2B). The induction of NPQ of Chl fluorescence depends on the level of acidification of the thylakoid lumen [29] and the redox state of the PQ pool [11,45], calculated by

NPQ = qL × [1 − Y(II)]/Y(II) × (Fv/Fm)/(1 − Fv/Fm) − 1

However, because of the second coefficient [1-Y(II)]/Y(II), the NPQ value increases as Y(II) decreases, as illustrated by the fact that the values of NPQ under 2 kPa O_2_ conditions were much higher than under 21 kPa O_2_ conditions during the illumination. Therefore, we needed to evaluate the acidification of the thylakoid lumen and the fluctuation of the net CO_2_ assimilation rate by different methods. Consequently, we measured the electrochromic shift (ECS) during the fluctuation.

**Figure 6:**
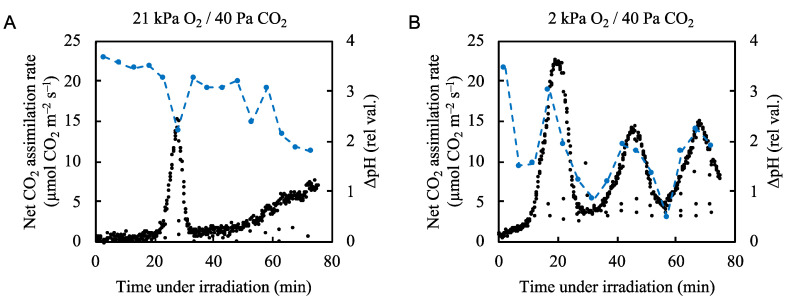
Effects of partial pressure of O_2_ on the relationship between the net CO_2_ assimilation rate and ΔpH across thylakoid membranes in rice leaves. The ΔpH across thylakoid membranes (blue circles) was measured simultaneously with the net CO_2_ assimilation rate (black circles) under (**A**) 21 kPa O_2_/40 Pa CO_2_ and (**B**) 2 kPa O_2_/40 Pa CO_2_ conditions. Actinic light (700 µmol photons m^–2^ s^–1^) was turned on at 0 min. Dashed blue lines in the graph were arbitrarily drawn to indicate the trend of the data.

We plotted ∆pH against the net CO_2_ assimilation rate under both normal and low-oxygen conditions (Figure 6, Supplementary Figure S11). Under normal atmospheric conditions ∆pH rapidly formed upon illumination with actinic light (Figure 6A). ∆pH decreased as the net CO_2_ assimilation rate increased and formed a small trough as the net CO_2_ assimilation rate peaked. The ∆pH level generally trended downward as the net CO_2_ assimilation rate trended upward. Under low oxygen conditions, the ∆pH level rapidly increased upon illumination but began to fluctuate along with the net CO_2_ assimilation rate, showing similar peaks and troughs (Figure 6B). These results indicate that photosynthetic linear electron transport, driven by both the CO_2_ assimilation and photorespiration, induces ∆pH formation across the thylakoid membranes. At the start of illumination with actinic light, we detected large increases in ∆pH even under low oxygen conditions, where the small Y(II) value that was not accounted for by photorespiration was detected (Figure 2B). These data suggest that an alternative electron flow to photorespiration contributed to the formation of ∆pH.

**Figure 7:**
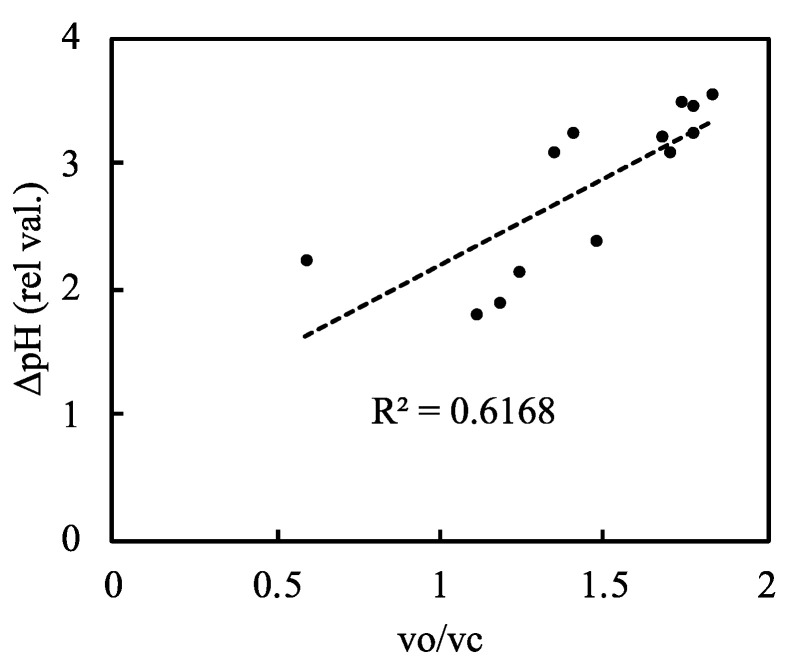
Relationship between the relative photorespiration activity (vo/vc) and ΔpH across the thylakoid membranes in rice leaves. The RuBP carboxylase reaction rate (vc) and the RuBP oxygenase reaction rate (vo) were estimated as described in the Materials and Methods. Data of vc and vo were simultaneously measured with ΔpH in Figure 6A. The dashed line in the graph is the regression line and the coefficient of determination was 0.6168 (*p* < 0.001).

We plotted ∆pH against the relative photorespiration rate (the vo/vc ratio) and found that ∆pH increased as the vo/vc ratio increased (Figure 7, Supplementary Figure S12). As the net CO_2_ assimilation rate decreased, photorespiration increased and induced the proton gradient across the thylakoid membranes. This suggests that photorespiration contributes to the oxidation of P700 via the induction of the proton gradient across thylakoid membranes.

## 3. Discussion

In this study, we attempted to elucidate the molecular mechanism of P700 oxidation in PSI during the intrinsic fluctuations of the net CO_2_ assimilation rate in rice plants. These fluctuations are driven by the periodic opening and closing of stomata that occurs in a somewhat random fashion (Figure 1A–D). We found that when CO_2_ assimilation is suppressed, photorespiration maintains the oxidation of P700 in PSI by inducing ∆pH across the thylakoid membranes. Lumen acidification suppresses the oxidation of the reduced PQ by the Cyt *b*_6_*f* complex, causing the reduction reaction of P700^+^ to become the rate-determining step in the P700 photo-oxidation/reduction cycle, and contributing to P700 oxidation. 

In general, upon exposure to environmental stresses such as drought, low or high temperatures, or high light levels, CO_2_ assimilation activity decreases and the amount of photon energy available exceeds that required to drive the CBB cycle [41]. Under these conditions, the photosynthetic electron transport system can begin to reduce O_2_ and produce ROS, especially at PSI of the thylakoid membranes, unless the accumulation of electrons is alleviated. This is known as the Mehler reaction, which is the primary reaction of the water–water cycle [41]. The electron carriers localized at the acceptor side of PSI (A_0_, A_1_, F_X_, F_A_/F_B_, and Fd) can easily reduce O_2_ to superoxide radicals [47,48,49]. However, the oxidation of P700 decreases the apparent quantum yield of PSI [50] and inhibits the accumulation of electrons on the acceptor side of PSI [12]. Furthermore, the oxidation of P700 maintains Fd in an oxidized state [42]. This is an important physiological function of the P700 oxidation system in photosynthetic organisms. Without it, PSI suffers from photoinhibition and loses its electron transport activity; photosynthetic organisms cannot survive under these circumstances [23]. In short, P700 oxidation is essential to suppress the production of ROS in PSI [2,42].

The reduction-induced suppression of electron flow also contributes to the oxidation of P700 in PSI and lumen acidification [2,32,33,51]. The suppression of the CBB cycle activity lowers the oxidation of P700 temporarily, with increases in the reduction ratios of Fd and PQ. The accumulation of reduced PQ inhibits the Q-cycle in the Cyt *b*_6_*f* complex, which down-regulates the electron flow from PQ to PC. Then, the reduction of P700^+^ by the reduced PC is suppressed and oxidized P700 accumulates. We can see the function of reduction-induced suppression of electron flow in the rice fluctuation situation under 2 kPa O_2_ conditions (Figure 3F, H and 6B). Under non-photorespiratory conditions (2 kPa O_2_/40 Pa CO_2_), both PC and P700 were rapidly oxidized as the net CO_2_ assimilation rate decreased after reaching its first peak (Figure 3). The acceleration of the CBB cycle simultaneously drives the photosynthetic linear electron flow with the formation of ∆pH across the thylakoid membranes (Figure 6B), contributing to the oxidation of P700 [2]. The rapid increases in the oxidation of both PC and P700 deviated from the increase in ∆pH. We found that the oxidation of both the reduced Fd and the reduced PQ was slower and they were maintained in a reduced state even after an increase in the net CO_2_ assimilation rate after reaching the first peak (Figure 6B). These findings indicate that the oxidation of both PC and P700 was induced by the reduction-induced suppression of electron flow [2,11,32,33]. 

Under 2 kPa O_2_ conditions, when photorespiration was suppressed the pattern of fluctuations in Y(II) deviated from the pattern formed by the net CO_2_ assimilation rate when the net CO_2_ assimilation rate was at its minimum (Figure 2B, Supplementary Figure S2). This suggests that an alternative electron flow to photorespiration functioned under these conditions, although it does not seem to be involved in mechanisms of photoprotection (Figure 3E–H). We also detected a rapid increase in ∆pH at the start of illumination with actinic light (Figure 6B). This suggests that the alternative electron flows that contributed to the formation of ΔpH would function in induction phases. There are many potential candidates for these alternative electron flows including S,N-assimilation, chloroplast respiration, and other pathways that consume reduced Fd [39,40,41,52]. There is a possibility that all of these candidates are involved in the excessive electron flux at PSII. The most plausible candidate for this system is the Mehler-ascorbate peroxidase (MAP) pathway (the water–water cycle) [41,53,54,55]. The MAP pathway drives the O_2_-dependent electron flow, which can function even at 2 kPa O_2_. This is similar to the flavodiiron (FLV)-dependent electron flow observed in cyanobacteria, green algae, mosses, ferns, and gymnosperms [13,23,56,57]. However, unlike the FLV-dependent electron flow, the magnitude of the electron flux of the MAP pathway is small. It remains to be determined whether the MAP pathway can function under 21 kPa O_2_/40 Pa CO_2_ conditions.

Fluctuations in the opening and closing of stomata have been observed as stomatal oscillations in many plant species [58,59,60]. Oscillatory transpiration is regulated by stomatal conductance, which senses the intercellular CO_2_ concentration and the leaf water status in order to maximize CO_2_ assimilation while minimizing water loss [60]. Under natural daylight conditions, the stomata oscillate with random frequency (Figure 1) [59]. These stomatal oscillations can continue for up to 10 h. Our results indicate that under natural field conditions, P700 oxidation is driven by photorespiration to suppress ROS production when CO_2_ assimilation is suppressed by stomatal closure in C3 plants.

In other land species, such as mosses, ferns and gymnosperms, another large alternative electron flow as well as photorespiration—FLV—is suggested to contribute to P700 oxidation [1,13,23,56,57]. It is not clear whether photorespiration also plays an important role in these plant species, or whether they show such large fluctuations in the opening and closing of stomata. It is probably the case that photorespiration can respond to the down-regulation of the CO_2_ assimilation efficiency faster and with reduced electron wastage than FLV because the primary reactions of both CO_2_ assimilation and photorespiration are mediated by the same enzyme, Rubisco.

C4 plants have no significant activity of photorespiration and no FLV protein [1,10,61,62,63]. Moreover, it has been shown that the alternative electron flows in C4 plants are negligible [1,10]. It was reported that maize also shows fluctuations in transpiration rate and stomatal conductance [58]. It remains to be elucidated how C4 plants cope with fluctuations in the opening and closing of stomata without photorespiration. However, it is possible that C4 plants benefit from the CO_2_ concentrating mechanism (CCM) [61] and can minimize the influence of the fluctuations of the stomatal aperture on the CO_2_ assimilation efficiency by maintaining a high partial pressure of CO_2_ around Rubisco.

## 4. Materials and Methods 

### 4.1. Plant Materials

Wild-type rice (*Oryza Sativa* L. cv. Nipponbare) plants were grown hydroponically in a controlled chamber (14 h light at 27 °C/10 h dark at 22 °C; light intensity 300–400 µmol photons m^−2^ s^−1^; relative humidity 50–60%). Two-week-old seedlings were planted in pots filled with the hydroponic solution described by Makino et al. [64]. The solution was renewed once a week and its concentration increased with plant age as described by Makino et al. [64]. For all experiments, we measured the second leaves from the tops of 6–8 week-old plants. 

### 4.2. Conditions during Measurements

We made measurements under two conditions: 21 kPa O_2_/40 Pa CO_2_ and 2 kPa O_2_/40 Pa CO_2_. While making measurements under these conditions, we adjusted the photosynthetic photon flux density to 700 µmol photons m^−2^ s^−1^, the relative humidity to 60%, the leaf temperature to 25 ± 1 °C, and the partial pressure of CO_2_ to 40 Pa.

### 4.3. Measurement of Changes in the Partial Pressure of CO_2_ and H_2_O to Measure Net CO_2_ Assimilation and Transpiration Rate

Changes in the partial pressure of CO_2_ and H_2_O were measured in a 3010-Dual gas exchange chamber (Heinz Walz, Effeltrich, Germany) using either a GFS-3000 (Heinz Walz, Effeltrich, Germany) or an LI-7000 (LI-COR, Lincoln, Nebraska, USA) gas analyzer. The former was used simultaneously with the Dual-KLAS/NIR system (Heinz Walz, Effeltrich, Germany) and the latter was used with the KLAS-100 system (Heinz Walz, Effeltrich, Germany), which are described below. In the measuring chamber, the ambient air was saturated with water vapor at 14.0 ± 0.1 °C and the cuvette temperature was maintained at 25 ± 0.5 °C, making the relative humidity 55–60%. We measured the transpiration rate, net CO_2_ exchange rate (A), and dark-adapted respiration rate (Rd), which was assumed to be equal to that under illumination. The gas exchange parameters were calculated according to the method of von Caemmerer [65].

### 4.4. Simultaneous Measurement of Oxidized P700 (P700^+^), Oxidized PC (PC^+^), Reduced Fd (Fd^−^) and Chlorophyll Fluorescence Using the Dual-KLAS/NIR

The redox states of the P700, PC, and Fd were measured using a Dual-KLAS/NIR instrument under two conditions (21 kPa O_2_/40 Pa CO_2_ and 2 kPa O_2_/40 Pa CO_2_). The Dual-KLAS/NIR measured the absorbance changes at four dual wavelengths (785–840 nm, 810–870 nm, 870–970 nm, and 795–970 nm). These signals were deconvoluted into the three signals that depend on PC^+^, P700^+^, and Fd^+^ by referring to a differential model plot as described by Klughammer et al., Kodota et al., and Shimakawa et al. [42,66,67] (Supplementary Figure S13, Supplementary Table S1). Before the measurements, we obtained the maximal oxidation/reduction levels of P700^+^, PC^+^, and Fd^−^ and we converted all signals into relative values. The redox levels under actinic light (640 nm, 700 µmol photons m^−2^ s^−1^) were determined using dirk interval relaxation kinetics (DIRK) analysis as described by Sacksteder and Kramer [68].

Chlorophyll fluorescence was also measured with the Dual-KLAS/NIR instrument. Weak measuring light (540 nm) was applied to leaves to obtain the basal level of chlorophyll fluorescence (Fo in the dark-adapted state, Fo’ under actinic light) and actinic light was applied to obtain the stationary level of fluorescence under actinic light (Fs). A short pulse of saturated light (300 ms, 20,000 µmol photons m^−2^ s^−1^) was applied to leaves to get the maximal fluorescence in the dark-adapted state (Fm) and under actinic light (Fm’). Using these values, we calculated the following parameters as described by Baker et al. [29]:
**terms****abbreviations****equations**The maximal quantum yield of PSIIFv/Fm(Fm − Fo)/FmThe effective quantum yield of PSIIY(II)(Fm’ − Fs)/Fm’The non-photochemical quenchingNPQ(Fm/Fm’) − 1The parameter reflecting the oxidation level of Q_A_, which is the primary quinone acceptor of PSIIqL(Fm’–Fs)/(Fm’ − Fo’)×(Fo − /Fs) or [Y(II)/(1 − Y(II))]×[(1 − (Fv/Fm))/(Fv/Fm)] ×(NPQ + 1) [45]

We evaluated CO_2_ assimilation and photorespiration activities using a previously-reported method [10,11,37,38]. The RuBP carboxylase reaction rate (vc) and RuBP oxygenation rate (vo) were calculated as follows:

vc = (1/6) × [ETR/2 + 4 × (A + Rd)](1)

vo = (1/6) × [ETR − 4 × (A + Rd)](2)

ETR (the electron transport rate in PSII) = Y(II) × the photosynthetic photon flux density × α, where α is the distribution ratio of photon energy to PSII. We used a value of 0.42 for α, as described by Wada et al. 2018 [69]. A is the net CO_2_ exchange rate and Rd is the respiration rate under actinic light, which was assumed to be equal to the dark-adapted respiration rate.

### 4.5. Simultaneous Measurement of the Proton Motive Force (pmf), the Proton Gradient (ΔpH) between the Lumen and Stroma, and the Chlorophyll Fluorescence Using the KLAS-100

The KLAS-100 instrument measured transmittances at 8 dual wavelengths: 505–520 nm, 520–534 nm, 540–545 nm, 545–549 nm, 549–553 nm, 553–559 nm, 559–563 nm, and 564–568 nm. These were deconvoluted into 7 signals representing c550, Cyt *b_559_*, Cyt *b_563_*, Cyt *f*, P515, the scattering signal, and zeaxanthin, based on a differential model plot obtained using the chloroplasts of spinach leaves [70,71]. The P515 is the electrochromic pigment absorbance shift at 505–520 nm. We used the same equipment to simultaneously measure chlorophyll fluorescence using a measuring light wavelength of 620 nm.

To obtain information about ∆pH, we applied 30 s of dark to the leaves according to the method of Klughammer et al. [72]. Each P515 signal was normalized by the change in width of P515 signals depending on the single charge separation when a single turnover (50 µs) was applied. We estimated the chlorophyll fluorescence parameters as described above. We applied a short pulse of saturated light 5 s before the 30 s of dark and repeated this treatment every 5 min. 

### 4.6. Statistical Treatments

For the data in Figure 4 and Figure 7 and Supplementary Figures S1, S2, S9 and S12, the coefficient of determination, which was equal to the square of the Pearson correlation coefficient of measured parameters, and the regression line were calculated or drawn using Microsoft Excel and JMP 14 (SAS Institute Japan, Tokyo, Japan).

## Supplementary Materials

The following are available online at https://www.mdpi.com/2223-7747/9/12/1761/s1. Figure S1, The correlation between the net CO_2_ assimilation rate and transpiration rate. Figure S2, The correlation between the electron transport rate and the net CO_2_ assimilation rate. Figure S3, The correlations between Y(II) and NPQ, and Y(II) and ΔpH. Figure S4, Data of Y(II) with the fluctuation of CO_2_ assimilation rate in each condition from two more individuals of plants. Figure S5, Data of the oxidation ratio of plastocyanin with the fluctuation of CO_2_ assimilation rate in each condition from two more individuals of plants. Figure S6, Data of the reduction ratio of ferredoxin with the fluctuation of CO_2_ assimilation rate in each condition from two more individuals of plants. Figure S7, Data of the oxidation ratio of P700 with the fluctuation of CO_2_ assimilation rate in each condition from two more individuals of plants. Figure S8, Data of the reduction level of Q_A_ (1-qL) with the fluctuation of CO_2_ assimilation rate in each condition from two more individuals of plants. Figure S9, Data of the correlation between the relative photorespiration activity (vo/vc) and the oxidation ratio of P700 during the fluctuation of CO_2_ assimilation rate in atmospheric condition from two more individuals of plants. Figure S10, Data of the non-photochemical quenching (NPQ) with the fluctuation of CO_2_ assimilation rate in each condition from two more individuals of plants. Figure S11, Data of ΔpH across the thylakoid membranes while the fluctuation of CO_2_ assimilation rate in each condition from two more individuals of plants. Figure S12, Data of the correlation between the relative photorespiration activity (vo/vc) and ΔpH across the thylakoid membranes during the fluctuation of CO_2_ assimilation rate in atmospheric condition from two more individuals of plants. Figure S13, Deferential Model Plots for P700, plastocyanin, and ferredoxin. Table S1, Examples of DMP values in rice leaves.
