# Supplementary material for: Intrinsic Fluctuations in Transpiration Induce Photorespiration to Oxidize P700 in Photosystem I
Source: Plants (Basel). 2020 Dec 12;9(12):1761. doi: 10.3390/plants9121761 (PMC7763966; doi:10.3390/plants9121761)
Supplement: Supplementary file 1 [file plants-09-01761-s001.pdf]

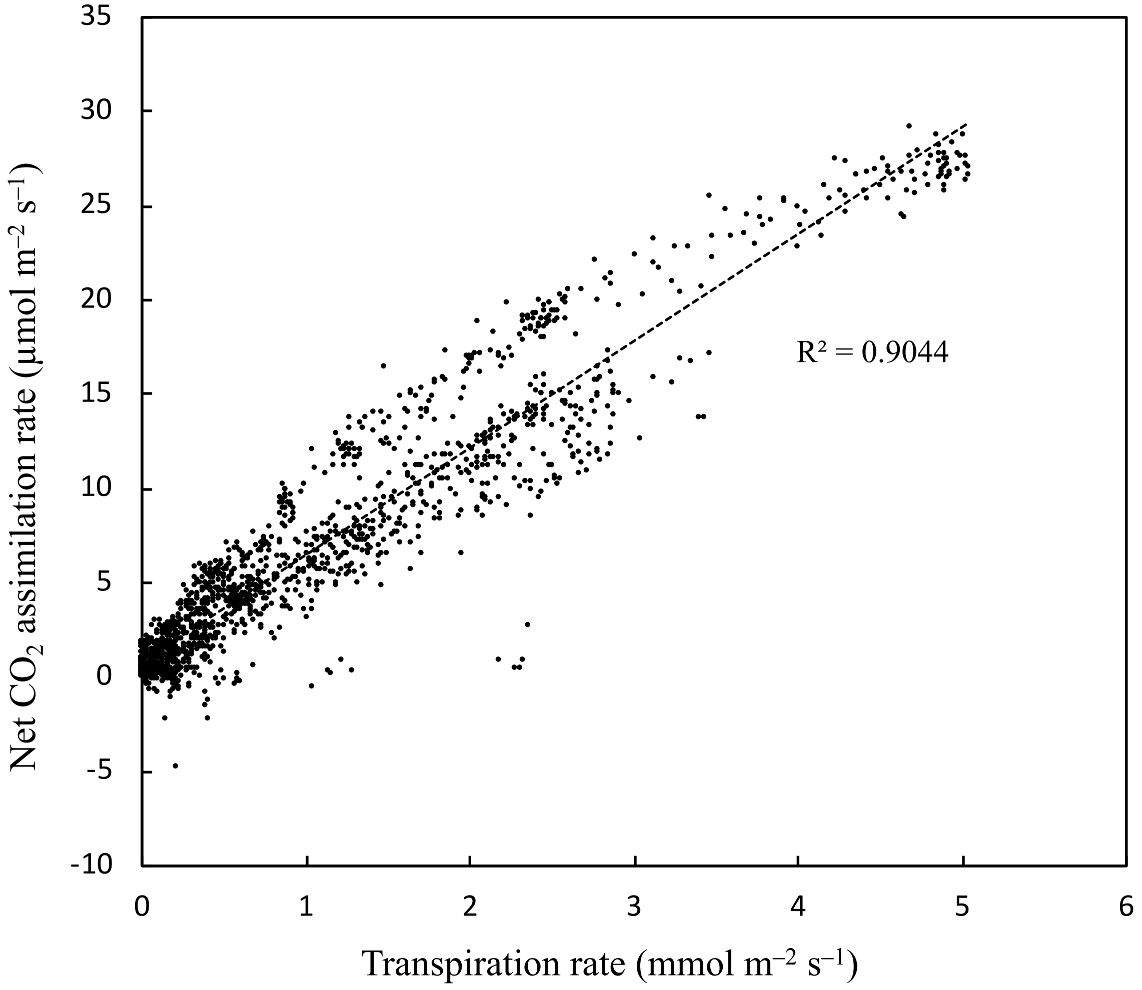

Supplemental Figure S1. The correlation between the net CO_2 assimilation rate and transpiration rate while the fluctuation under the illumination with actinic light (700 µmol photons m^{-2} s^{-1}) in atmospheric condition (21 kPa O_2/ 40 Pa CO_2). The data plots are taken from six independent measurements. The broken line shows the regression line and the coefficient of determination is 0.9044 ($p<0.001$) as shown in the plot.

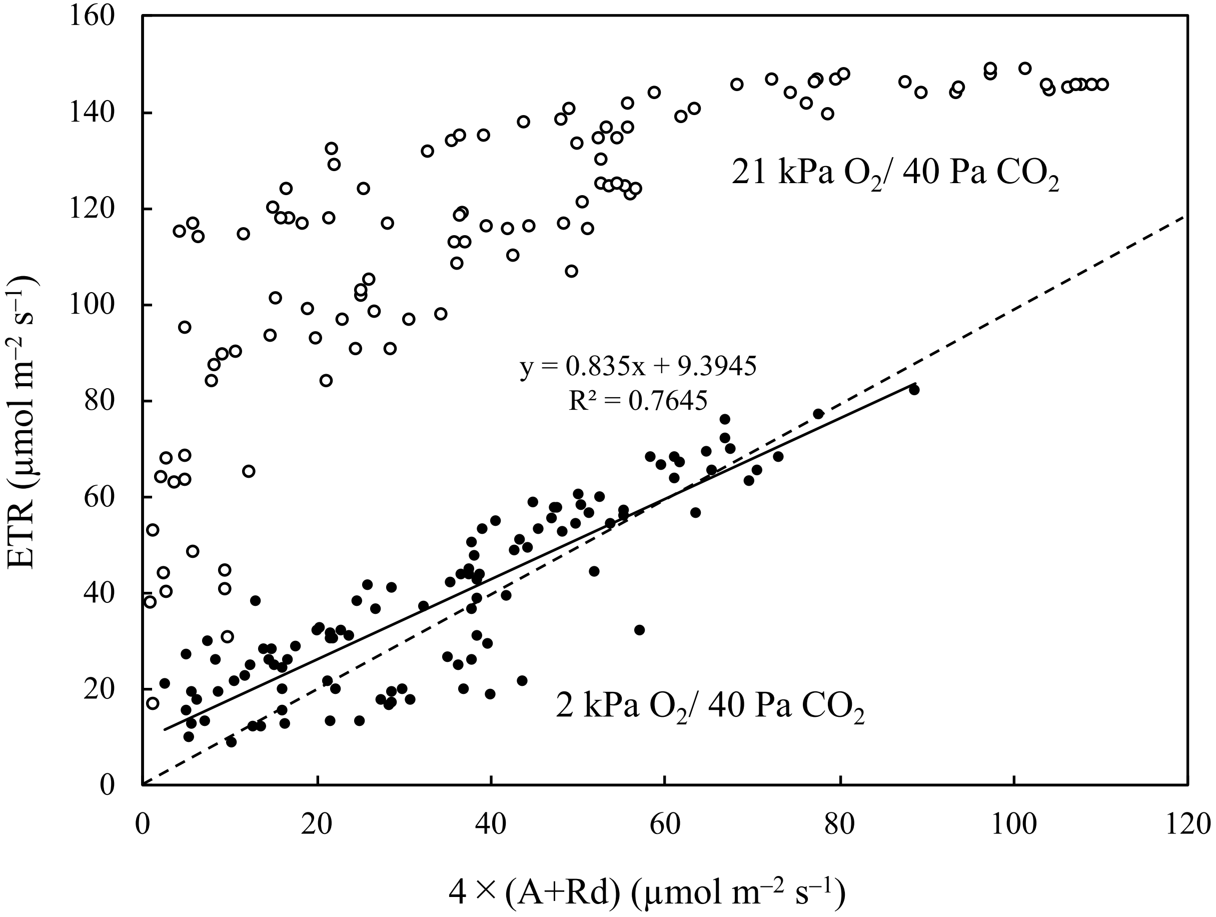

Supplemental Figure S2. Correlation between ETR and 4 × (A+Rd) in 21 kPa O_2/ 40 Pa CO_2 (open circles) and 2 kPa O_2/ 40 Pa CO_2 (closed circles) conditions. The data plots includes the induction phases in each condition from 5 independent measurements. The broken line shows the 1:1 line of ETR and 4 × (A+Rd). The solid line shows the regression line in 2 kPa O_2 condition. The intercept of it is 9.3945 ($p<0.001$) and the coefficient of determination is 0.7645 ($p<0.001$). ETR was calculated by the following equation; ETR = Y(II) × PPFD × α. PPFD was 700 μmol photons m^{-2} s^{-1} and the value of α was 0.42 as previous research by Wada et al. 2018.

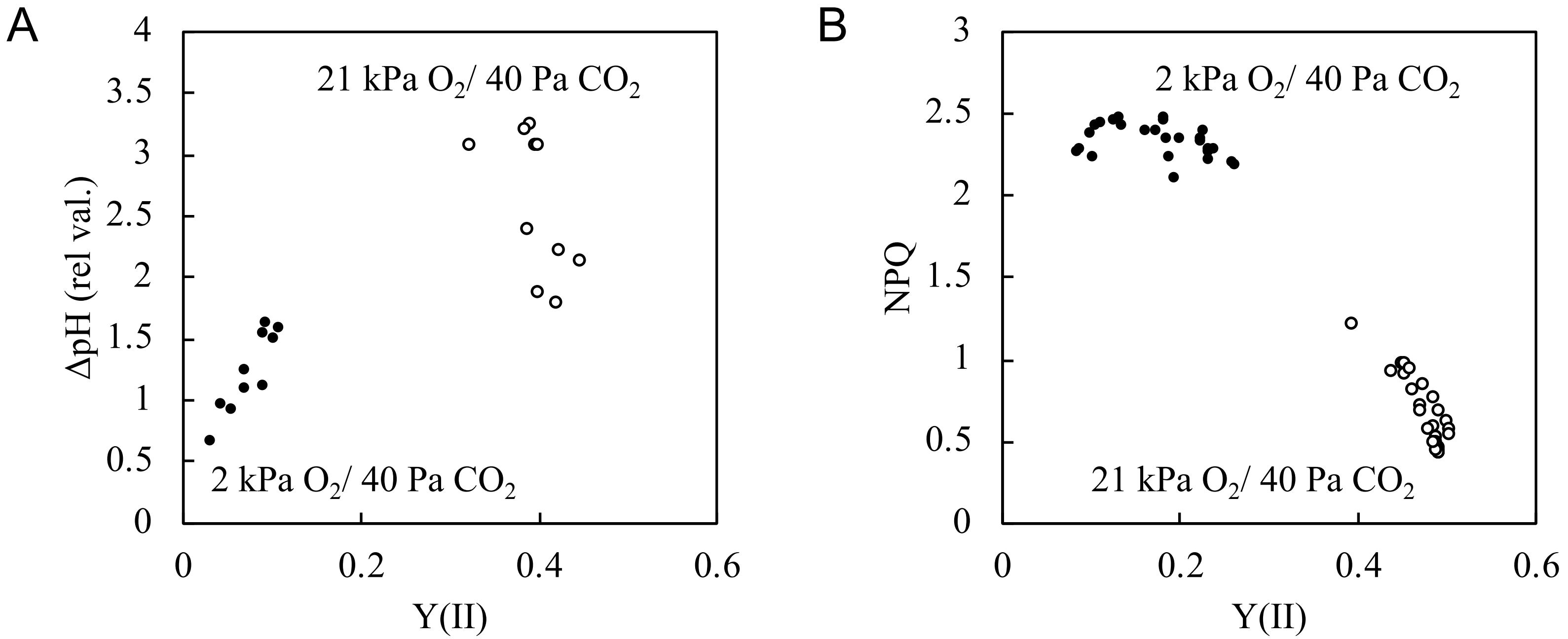

Supplemental Figure S3. Correlation between Y(II) and ΔpH across the thylakoid membranes (A), and Y(II) and the non-photochemical quenching (NPQ) (B) while the fluctuation of the net CO_2 assimilation rate under steady actinic light (700 µmol m^{-2} s^{-1}) at 21 kPa O_2/ 40 Pa CO_2 (open circles) and 2 kPa O_2/ 40 Pa CO_2 (closed circles). Data plots in A are the same as Figure 6 and those in B are the same as Figure 5. The induction phases in each measurement are removed from the data plots.

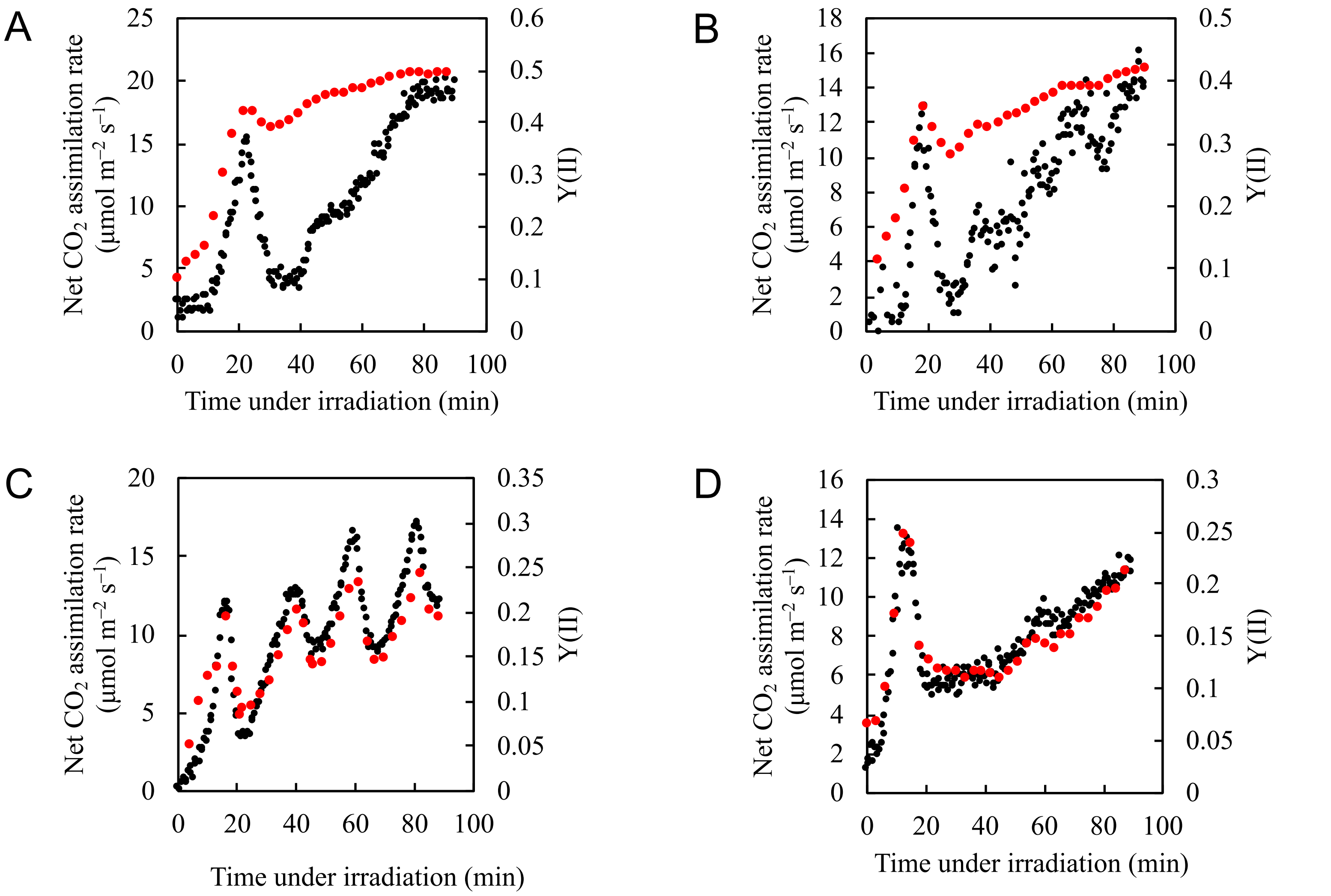

Supplemental Figure S4. Data of the effective quantum yield of PSII [Y(II)] (red circles) with the fluctuation of the net CO_2 assimilation rate (black circles) under the steady light (700 µmol photons m^{-2} s^{-1} of the light intensity) from another two individuals under 21 kPa O_2 (A,B) and 2 kPa O_2 (C,D) conditions.

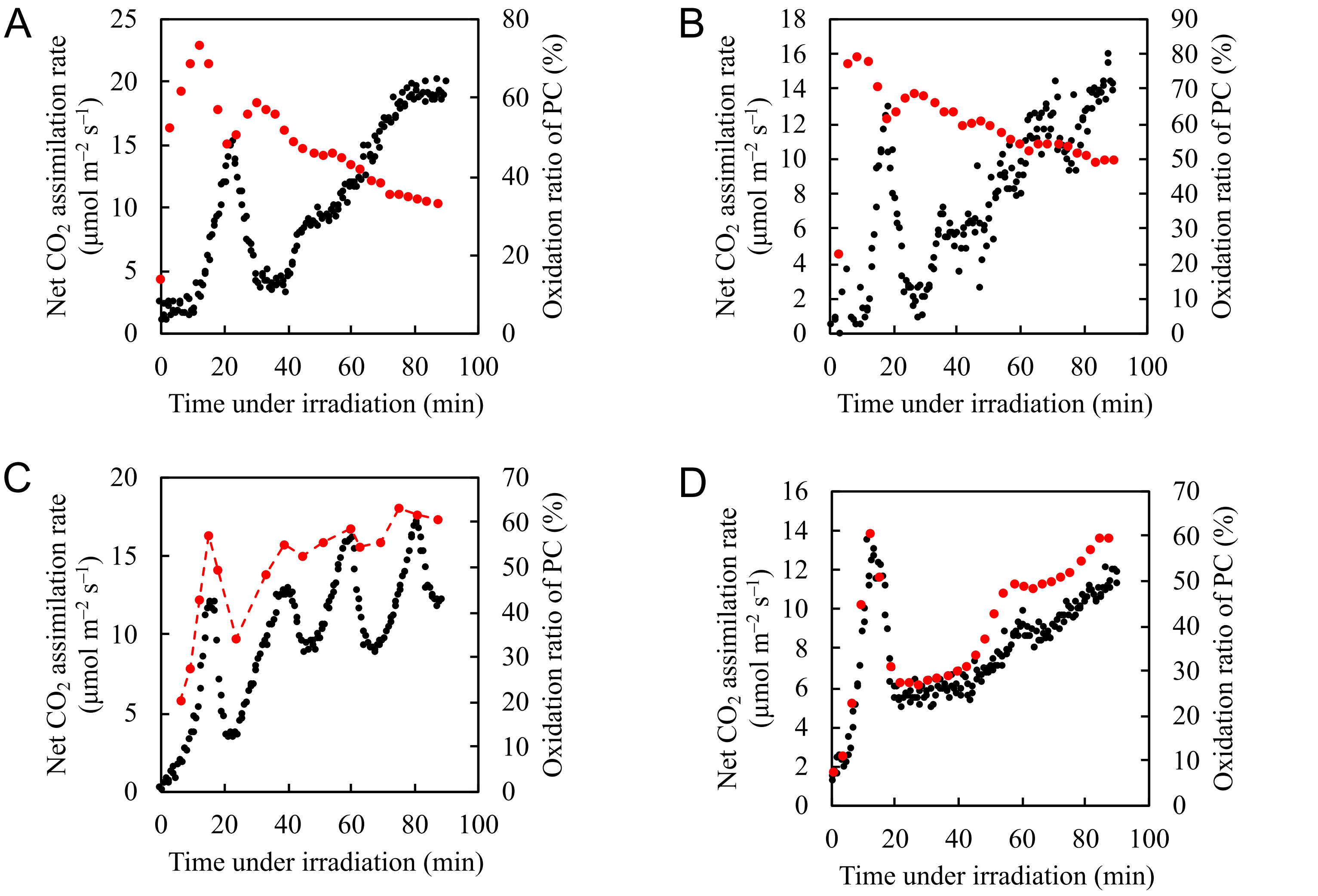

Supplemental Figure S5. Data of the oxidation ratio of plastocyanin (PC) (red circles) with the fluctuation of the net CO_2 assimilation rate (black circles) under the steady light (700 μmol photons m^{-2} s^{-1} of the light intensity) from another two individuals under 21 kPa O_2 (A,B) and 2 kPa O_2 (C,D) conditions.

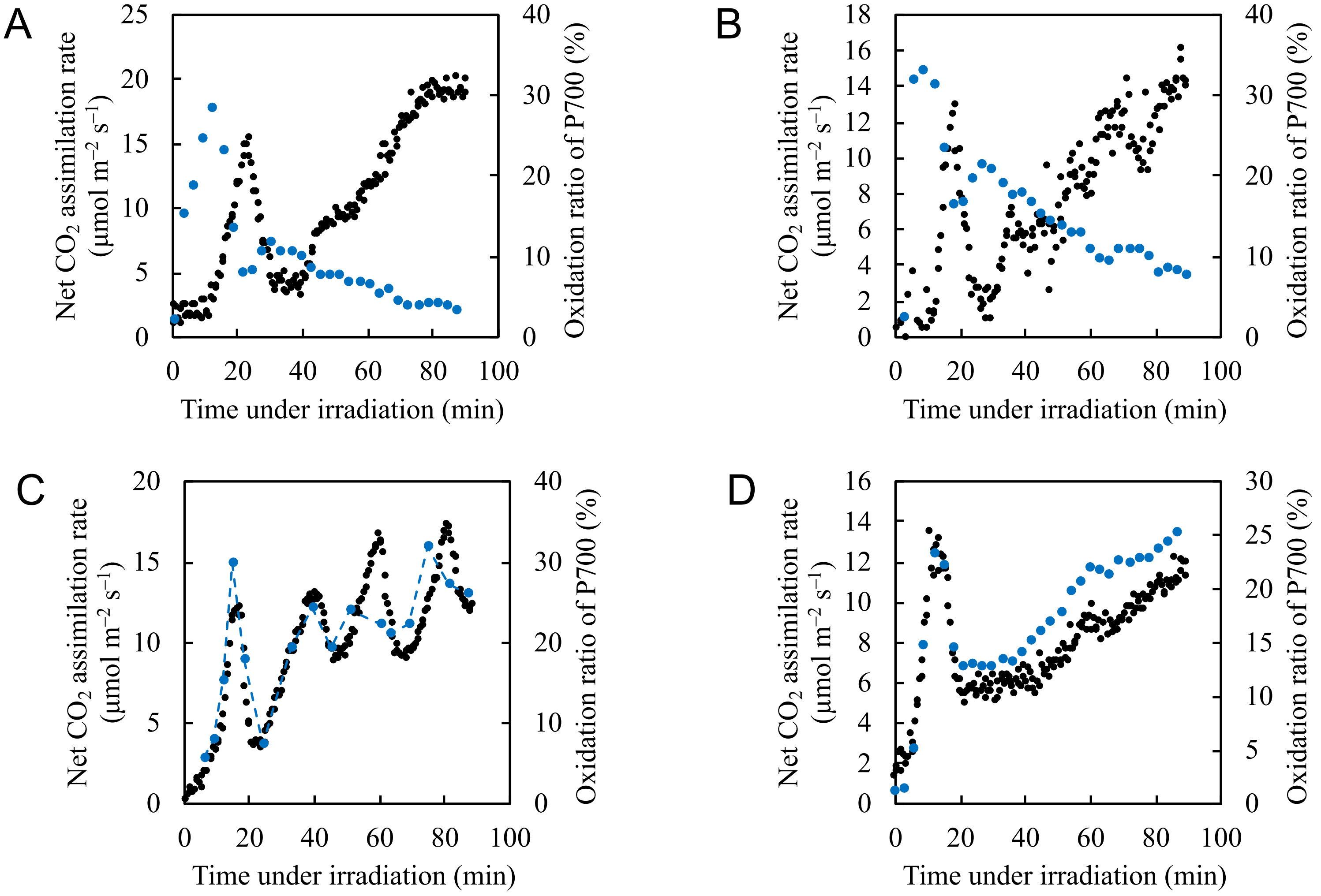

Supplemental Figure S6. Data of the oxidation ratio of the reaction center chlorophyll of PSI (P700) (blue circles) with the fluctuation of the net CO_2 assimilation rate (black circles) under the steady light (700 μmol photons m^{-2} s^{-1} of the light intensity) from another two individuals under 21 kPa O_2 (A,B) and 2 kPa O_2 (C,D) conditions.

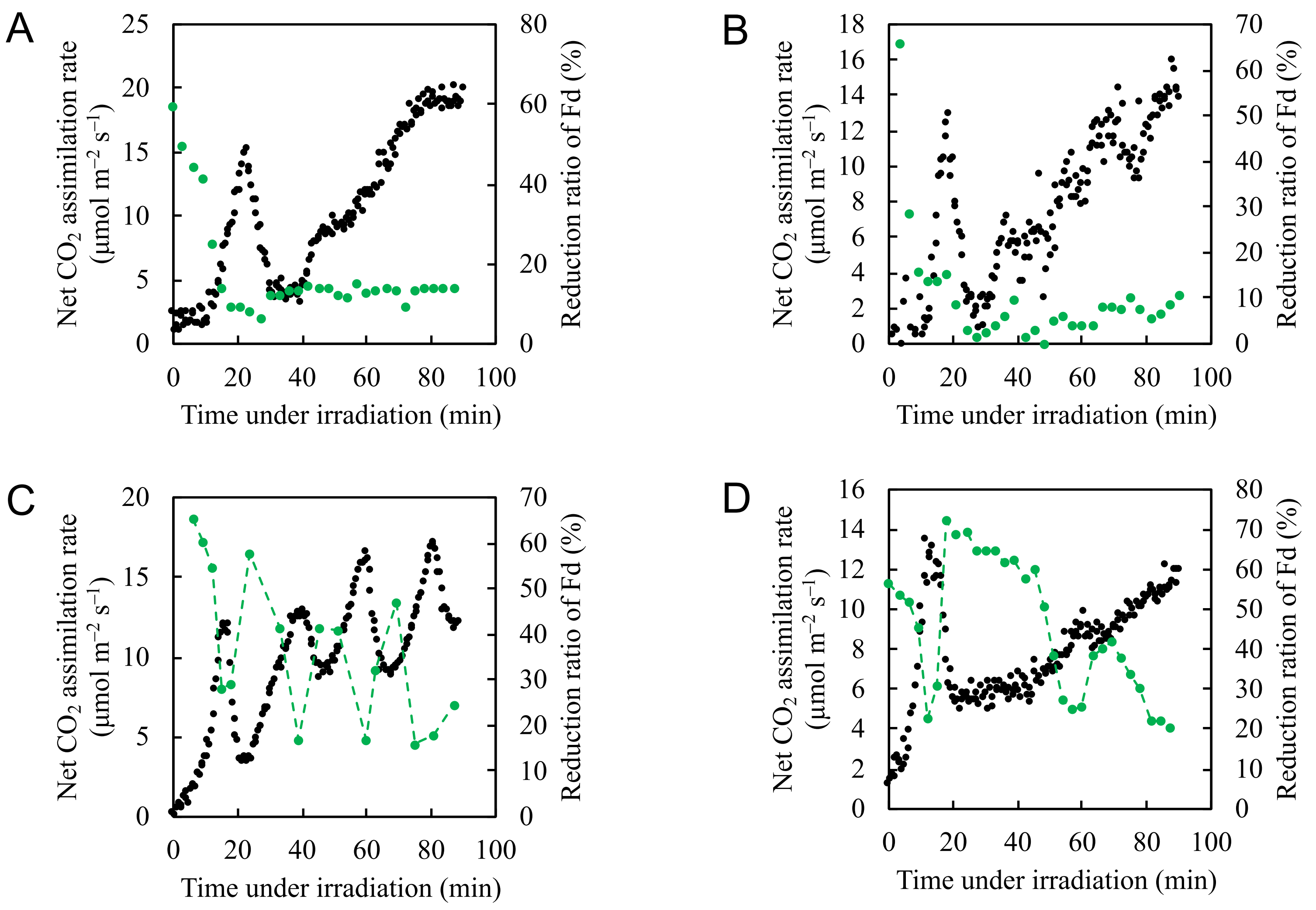

Supplemental Figure S7. Data of the reduction ratio of ferredoxin (Fd) (green circles) with the fluctuation of the net CO_2 assimilation rate (black circles) under the steady light (700 µmol photons m^{-2} s^{-1} of the light intensity) from another two individuals under 21 kPa O_2 (A,B) and 2 kPa O_2 (C,D) conditions.

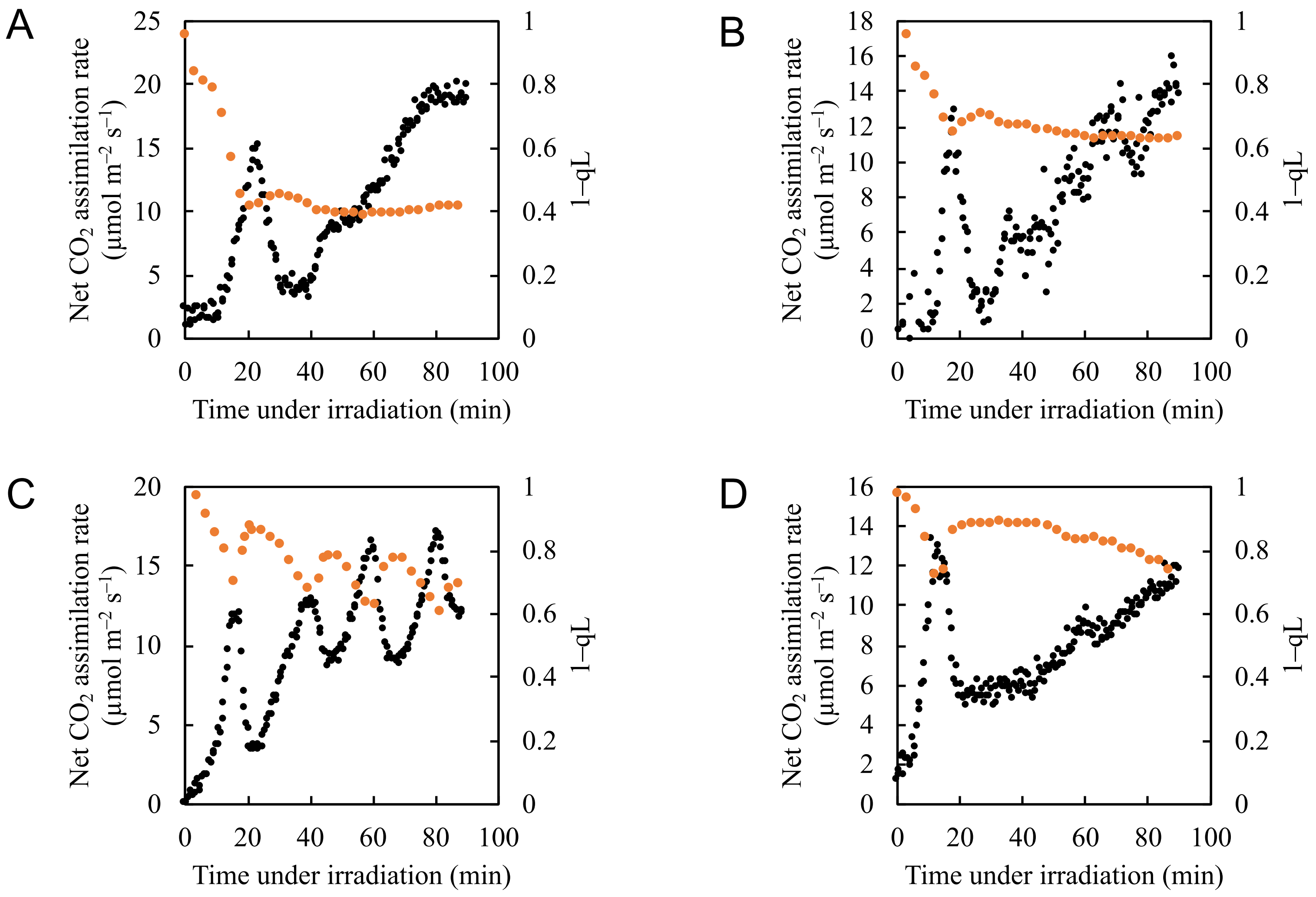

Supplemental Figure S8. Data of the reduction ratio of Q_A (1–qL) (orange circles) with the fluctuation of the net CO_2 assimilation rate (black circles) under the steady light (700 µmol photons m^{-2} s^{-1} of the light intensity) from another individuals under 21 kPa O_2 (A,B) and 2 kPa O_2 (C,D) conditions.

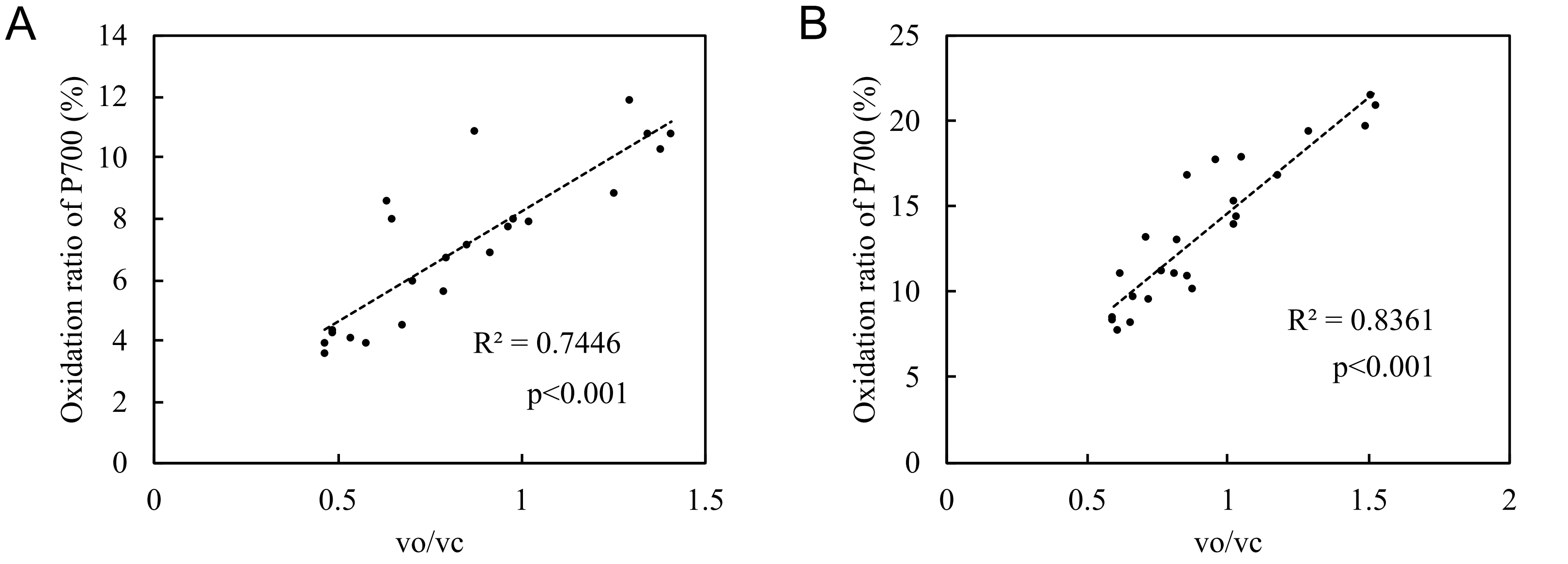

Supplemental Figure S9. Data of the correlations between the relative photorespiration activity (vo/vc) and oxidation ratio of P700 under the steady light (700 µmol photons m^{-2} s^{-1} of the light intensity) from another two individuals under 21 kPa O_2/ 40 Pa CO_2 condition. The data plots in A are from supplemental Figure S3A and B from supplemental Figure S3B. The broken lines show the regression line and the coefficient of determination are shown in each plot. The induction phases in each measurement are avoided from the data plots.

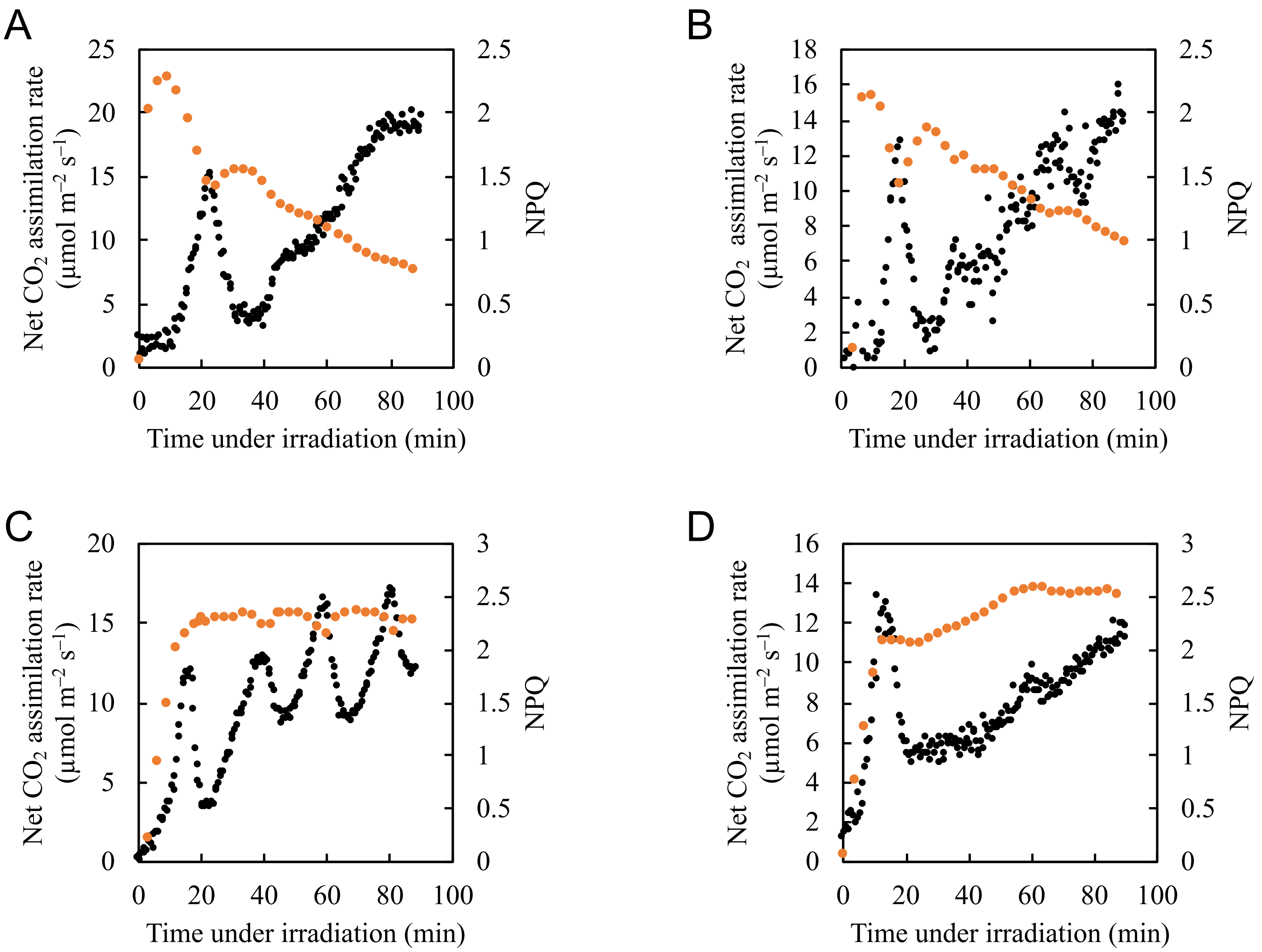

Supplemental Figure S10. Data of the non-photochemical quenching (NPQ) (orange circles) with the fluctuation of the net CO_2 assimilation rate (black circles) under the steady light (700 µmol photons m^{-2} s^{-1} of the light intensity) from another two individuals under 21 kPa O_2 (A,B) and 2 kPa O_2 (C,D) conditions.

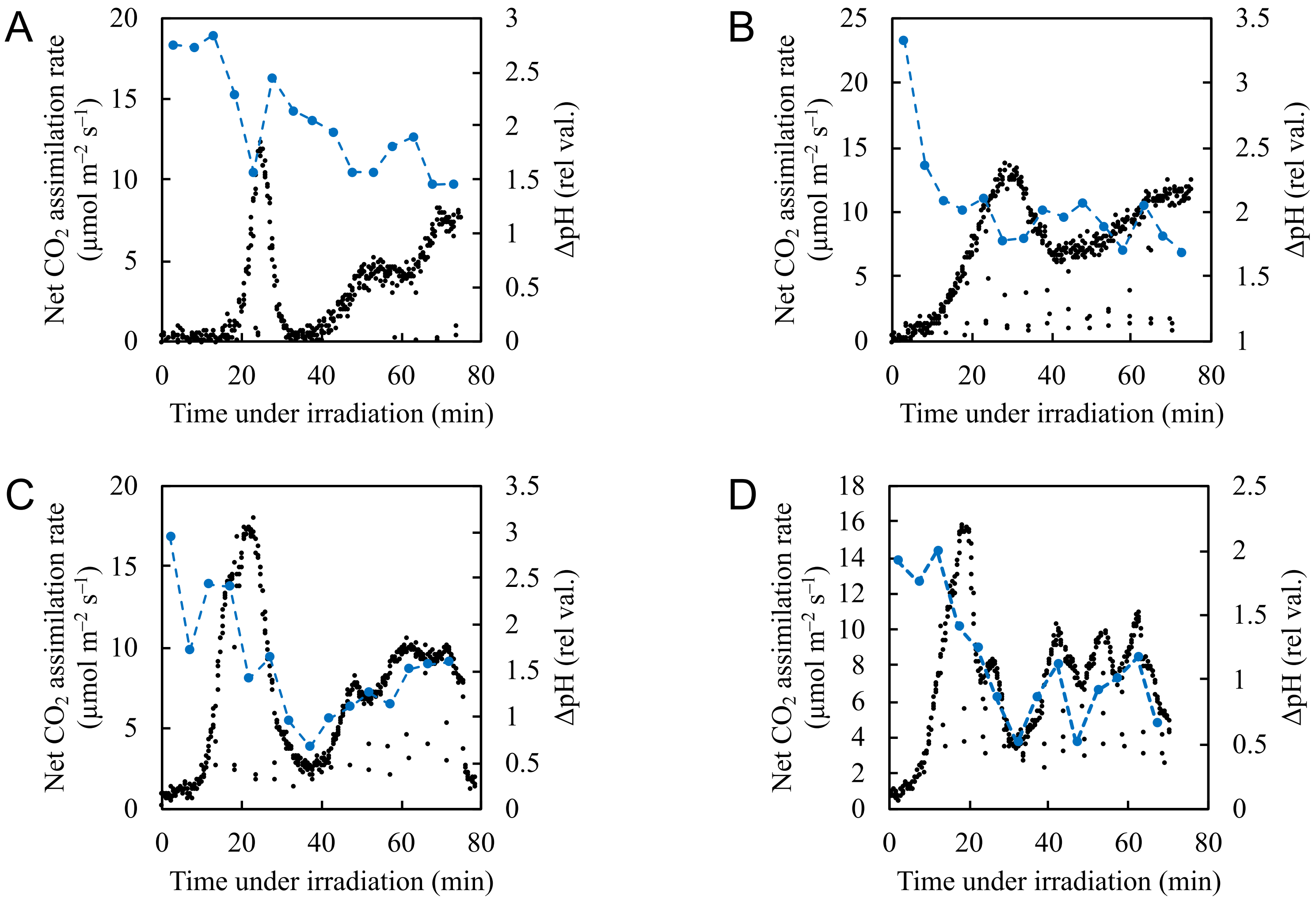

Supplemental Figure S11. Data of the proton gradient across thylakoid membranes (ΔpH) (orange circles) with the fluctuation of the net CO_2 assimilation rate (black circles) under the steady light (700 μmol photons m^{-2} s^{-1} of the light intensity) from another two individuals under 21 kPa O_2 (A,B) and 2 kPa O_2 (C,D) conditions.

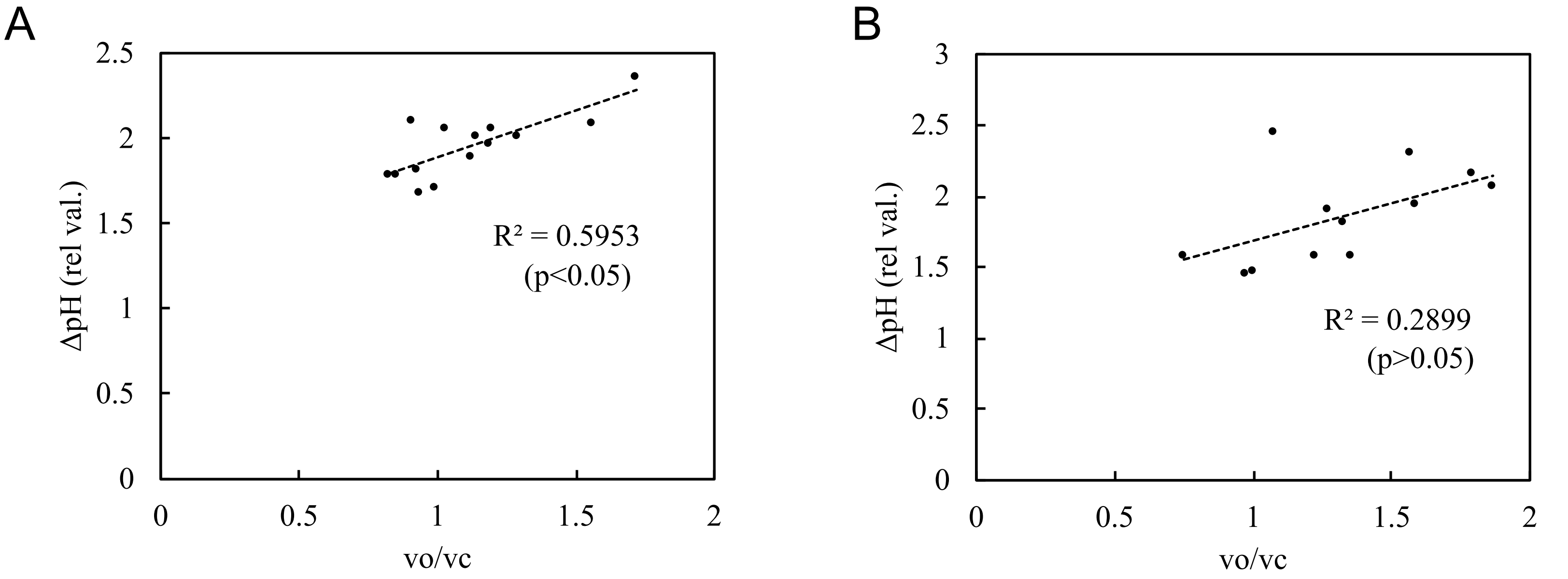

Supplemental Figure S12. Data of the correlations between the relative photorespiration activity (vo/vc) and proton gradient across the thylakoid membranes (ΔpH) while the fluctuation of the net CO_2 assimilation rate under the steady light (700 µmol photons m^{-2} s^{-1} of the light intensity) from another two individuals under 21 kPa O_2/ 40 Pa CO_2 condition. The data plots in A are from supplemental Figure S10A and those in B are from supplemental Figure S10B. The broken lines show the regression line and the coefficient of determination are shown in each plot. The induction phases in each measurement are avoided from the data plots.

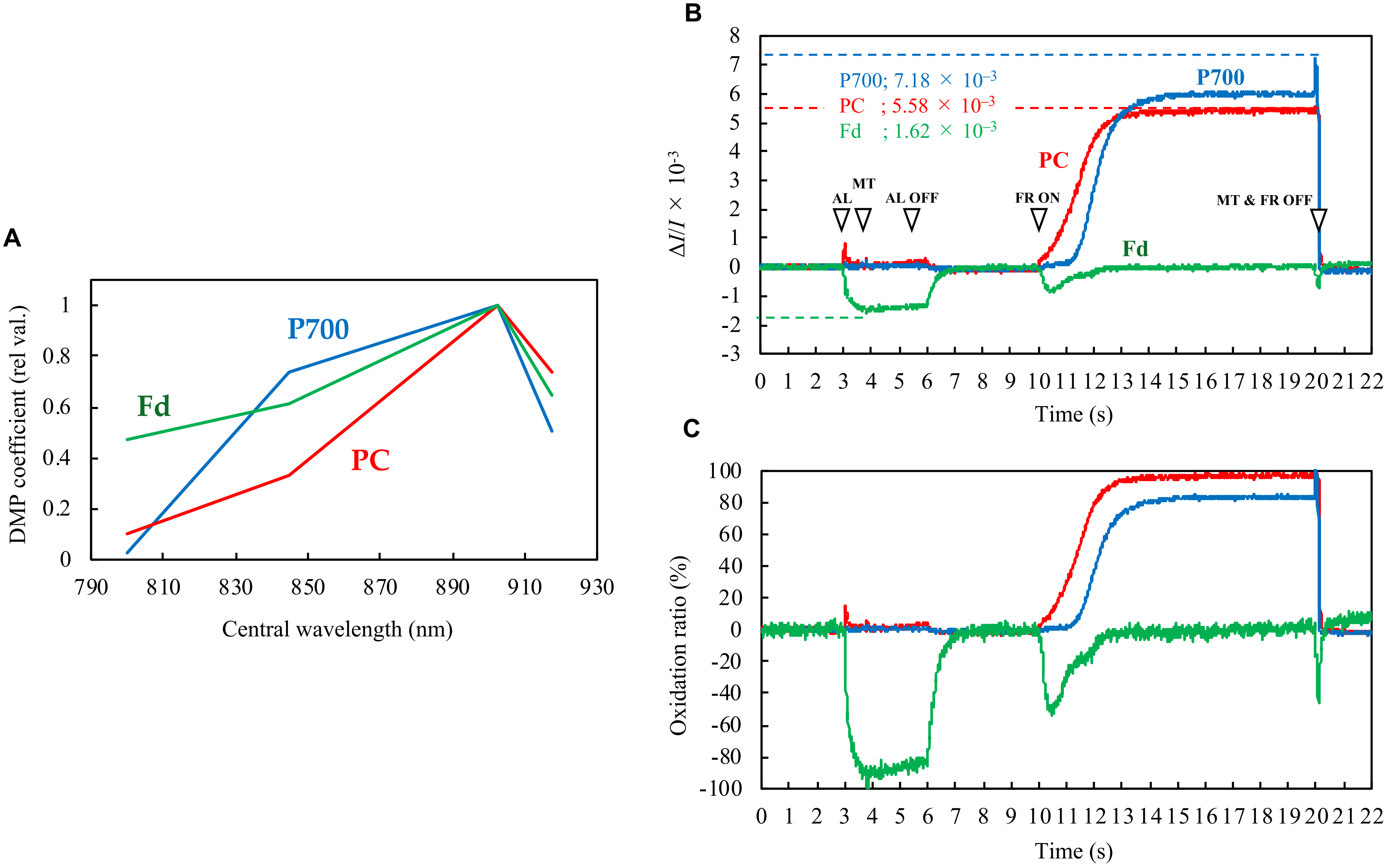

Supplemental Figure S13. Deferential Model Plots for P700, plastocyanin, and ferredoxin. (A) Examples of Deferential Model Plots (DMPs) for P700 (blue line), Fd (green line), and PC (red line). The DMPs were taken from rice leaves before the measurements were performed as described by Klughammer et al. (2016). Four dual wavelength signals were deconvoluted into the three signals representing P700, Fd, and PC. (B) To determine the relative amplitudes of the 100% reduction/oxidation changes, rice leaves were illuminated as follows. After starting to log the signals at 0 s, the leaves were illuminated with actinic light (AL; 630nm, 300 μmol photons m^{-2} s^{-1}) at 3 s. Then, multiple turnovers (MT; 10,000 μmol photons m^{-2} s^{-1}) were applied at 3.8 s to get the Fd 100% reduction change.

(**Supplemental Figure S13. continued**) AL was turned off at 6 s. Illumination with far red light (FR; 740 nm, 360 μmol photons m^{-2} s^{-1}) was applied from 10 s to 20 s. To determine the absorbance changes at 100% oxidation of PC and P700, MT was applied again at 20 s, and FR was turned off at 20 s. (C) From the data in B, the relative amplitudes of the 100% redox changes were determined: 7.18×10^{-3} for 100% oxidized P700, 5.58×10^{-3} for 100% oxidized PC, and 1.62×10^{-3} for 100% reduced Fd. According to these values, all signals were converted to % redox change scales. The right vertical axis shows the oxidation percentage, and negative values indicate reduction levels.

Supplemental Table S1. Examples of DMP values in rice leaves. Examples are derived from the specific absorbance changes by P700, PC and Fd. Data are from the DMPs shown in Supplementary Figure S1. Each value is normalized by the transmittance changes of the dual wavelengths in the range 795–970 nm.

	P700	Fd	PC
$\Delta I/I$ (785-840 nm)<br>Central value; 812.5 nm	0.0266	0.4721	0.1016
$\Delta I/I$ (810-870 nm)<br>Central value; 840.0 nm	0.7407	0.6107	0.3303
$\Delta I/I$ (795-970 nm)<br>Central value; 882.5 nm	1.0000	1.0000	1.0000
$\Delta I/I$ (870-970 nm)<br>Central value; 920.0 nm	0.5020	0.6480	0.7354
